# Targeting the Gut Microbiota and Host Immunity with a *Bacilli*-Species Probiotic during Antibiotic Exposure in Mice

**DOI:** 10.3390/microorganisms10061178

**Published:** 2022-06-08

**Authors:** David Shapiro, Fatemeh Ramezani Kapourchali, Anthony Santilli, Yingchun Han, Gail A. M. Cresci

**Affiliations:** 1 Department of Inflammation and Immunity, Cleveland Clinic, Cleveland, OH 44195, USA; shapird2@ccf.org (D.S.); ramezanik.fatemeh@gmail.com (F.R.K.); santila@ccf.org (A.S.); hany2@ccf.org (Y.H.); 2 Department of Pediatric Gastroenterology, Cleveland Clinic, Cleveland, OH 44195, USA; 3 Center for Human Nutrition, Cleveland Clinic, Cleveland, OH 44195, USA

**Keywords:** antibiotics, probiotic, retinoic acid, gut microbiome, nutrition immunity, intestine

## Abstract

Antibiotic therapy is necessary for the treatment of bacterial infections; however, it can also disrupt the balance and function of commensal gut microbes and negatively affect the host. Probiotics have been tested as a means to counteract the negative effects of antibiotic therapy, but many probiotics are also likely destroyed by antibiotics when taken together. Here we aimed to test the efficacy of a non-pathogenic spore-forming *Bacillus*-species containing a probiotic blend provided during antibiotic therapy on host immune defenses in mice. Mice were exposed to antibiotics and supplemented with or without the probiotic blend and compared to control mice. Fecal and cecal contents were analyzed for gut microbes, and intestinal tissue was tested for the expression of key enzymes involved in vitamin A metabolism, serum amyloid A, and inflammatory markers in the intestine. The probiotic blend protected against antibiotic-induced overgrowth of gram-negative bacteria and gammaproteobacteria in the cecum which correlated with host immune responses. Regional responses in mRNA expression of enzymes involved with vitamin A metabolism occurred between antibiotic groups, and intestinal inflammatory markers were mitigated with the probiotic blend. These data suggest prophylactic supplementation with a spore-forming *Bacillus*-containing probiotic may protect against antibiotic-induced dysregulation of host immune responses.

## 1. Introduction

Comprised of trillions of microbes, the gut microbiota has co-evolved along with the gut to create a mutualistic relationship with the host centered on gut immune responses and tolerance [1]. A variety of human diseases are associated with an imbalance in gut microbial composition and function often termed “gut dysbiosis”. Gut dysbiosis is characterized as expanded opportunistic microbial densities at the expense of reduced commensal microbes [2]. In order to prevent gut dysbiosis and maintain homeostasis between gut microbes and the host, the host employs several immune-mediated mechanisms. These include the release of secretory Immunoglobulin A (sIgA), antimicrobial peptides (AMPs), and downregulation of key nutrients desired by pathogens, such as iron. Secreted by B cells into the mucus layer and lumen of the intestine, sIgA binds to many commensal and pathogenic bacteria and their toxins to prevent their translocation from the gut lumen [3]. Vitamin A, provided from the diet in the form of carotenoids or retinyl esters, is also central to gut immune homeostasis where it coordinates both innate and adaptive immune responses. All-trans retinoic acid (*at*RA) is a potent active metabolite of vitamin A that functions as a ligand for the retinoic acid receptor (RAR). Under normal conditions the commensal bacteria in the gut regulate *at*RA production by controlling the expression of retinol dehydrogenase 7 (RDH7), the enzyme that converts retinol to retinal, which is ultimately converted into *at*RA. Controlling the concentration of *at*RA in the gut is vital as it can both prevent and promote infection. All-trans retinoic acid can promote IL-22 mediated antimicrobial responses, and following treatment with antibiotics certain opportunistic bacteria (e.g., Proteobacteria) can exploit this system and induce the overexpression RDH7 and increase *at*RA production [4]. Consequently, the IL-22 mediated antimicrobial response diminishes commensals which allows the pathogens to proliferate [4]. Conversely, *at*RA promotes B cell class switching to IgA-secreting plasma cells which protects the host against infections. Antibiotic therapy decreases gut microbial abundance and diversity and enhances opportunistic bacteria expansion and subsequent immune dysregulation and inflammation [5,6]. Antibiotic administration can also decrease circulating sIgA, increasing the host’s vulnerability to pathogenic infection [7].

All trans retinoic acid is also integral in the regulation of Serum Amyloid A (SAA), an acute phase inflammatory protein. Serum amyloid A (−1,2,3) bind retinol with high affinity, and are upregulated in the intestine and liver where they play a vital immunomodulatory and anti-inflammatory role during infection [8]. SAA is responsible for CD4^+^ T cell homing and can directly cause the induction of TH17 cells in response to pathogens [8,9]. The *at*RA receptor RARβ directly activates SAA expression in response *at*RA binding and regulates the expression of IL-17 by TH17 cells [8].

Antibiotics are prescribed at high rates in the hospital and ambulatory care setting, and in the United States at least 28% of these are deemed unnecessary [10]. Clindamycin is in a class of medications called lincomycin antibiotics used to treat anaerobic bacterial infections by slowing or stopping their growth. Vancomycin is in a class of medications called glycopeptide antibiotics and its oral administration kills gram-positive bacteria in the intestine, such as *Clostridioides difficile* (*C. difficile*). Mechanistically vancomycin inhibits bacterial cell wall formation and has little effect on spore germination [11]. Antibiotic-induced gut microbial disturbance can result in many negative effects including diarrhea and pathogen colonization, therefore use of probiotics as a means to protect against these effects has been investigated [12]. Effectiveness of probiotic therapy depends on multiple factors including the probiotic strain’s survivability in the gut lumen and against antibiotics, as well as its mechanism of action. As many probiotics in their vegetative form are readily destroyed by antibiotics, a non-pathogenic spore-forming *Bacillus* species-containing probiotic may be beneficial. Bacilli are gram-positive, aerobic, spore-forming bacteria that are widely spread in our environment, and have better survivability and stability in the gut due to their spore-forming abilities [13]. Bacilli can survive passage through the stomach in the form of spores, germinating and proliferating in the proximal and distal small intestine within 18–24 h of delivery [14]. Following germination *Bacillus* bacteria are either destroyed or travel to the colon and sporulate since germination is unlikely due to the nutrient deficient and other hostile environmental factors [13]. Owing to the enhanced survivability reasons of *Bacillus* species, we tested the notion that a blend of spore-forming *Bacillus* species would provide a protective host effect in mice treated with antibiotics. We previously reported a *Bacillus*-containing probiotic blend containing *B. licheniformis*, *B. indicus HU36™*, *B. subtilis HU58™*, *B. clausii*, and *B. coagulans*, preserves immune responses and protects intestinal integrity in mice exposed to *C. difficile* colonization [15]. Of particular interest to this project is that the species *B. Indicus HU36™* has been reported to produce carotenoids including lycopene, astaxanthin, lutein, and β-carotene [16]. This study aimed to test the efficacy of a *Bacillus*-species containing “probiotic blend” in protecting against antibiotic-induced gut dysbiosis and impaired host-derived gut immune defenses in mice.

## 2. Materials and Methods

### 2.1. Reagents

Animals were treated with a probiotic blend consisting of: *B. licheniformis*, *B. indicus HU36™*, *B. subtilis HU58™*, *B. clausii*, *B. coagulans* (MegaSporeBiotic*™*, Microbiome Labs, Saint Augustine, FL, USA), clindamycin (Fresenius Kabi, Lake Zurich, IL, USA), and vancomycin hydrochloride (Hospira, Inc., Lake Forest, IL, USA). Treatments were added onto mouse treats (Bacon Yummies Bio-Serv, Flemington, NJ, USA). Throughout the trial animals were fed sterile Teklad Global 18% Protein extruded rodent diet (Harlan Teklad, Madison, WI, USA). All quantitative real-time polymerase chain reaction (qRT-PCR) primers were obtained from Integrated DNA Technologies (Coralville, IA, USA). Intestinal sections were stained with rabbit anti-CD3 (Abcam, Cambridge, MA, USA), anti-rabbit Alexa 568 (Invitrogen, Eugene, OR, USA), and Anti-Dapi (Littleton, CO, USA).

### 2.2. Mouse Model

Female, CF-1 mice (10 weeks old) were purchased from Charles River Laboratories (Wilmington, MA, USA). All animal methods were approved by the Cleveland Clinic Institutional Animal Care and Use Committee. For the duration of the experiment, all mice were individually housed in microisolator cages and given sterile 2018 Teklad Global 18% Protein extruded rodent diet (Envigo). Mice were treat-trained with Bacon Yummies for four days to encourage consumption of treat within 20 min of its provision. From day five until trial end mice were treated with 80 mg of probiotic blend or 0.1 mL saline via mouse treat or oral gavage. Additionally, starting on day five animals were given subcutaneous injection of either 1.4 mg Clindamycin or a 0.1 mL saline, daily for three days. From day 12 until study end, mice received 2.25 mg vancomycin or 0.1 mL saline daily via treat or by oral gavage.

### 2.3. Selective Bacterial Plating

Agar plating for selective bacteria was performed on fresh fecal pellets collected throughout the trial, and cecal content immediately obtained following euthanasia. At specific time points fresh fecal pellets were collected. The mice were placed into new sterile cages and left for 1 h. After 1 h fecal pellets were taken from the cages using sterile forceps and the feces were placed in a pre-weighed 1.7 mL tubes. The tubes with the fecal pellets were weighed and immediately plated as previously described [17]. Briefly, fecal pellets and cecal content were suspended at a 10 mg/µL in sterile 1X PBS. The fecal suspension was then serially diluted and plated on Difco MacConkey Agar (BD, Franklin Lakes, NJ, USA) for enumeration of aerobic and facultative anaerobic gram-negative rods. To enumerate enterococcoci the dilutions were also plated on BBL Enterococcosel Agar (BD, Franklin Lakes, NJ, USA). The plates were then incubated aerobically at 37 °C and colonies were counted at 24 and 48 h. Data are presented as log_10_ Colony Forming Units (CFU) per gram of fecal or cecal content.

### 2.4. Fecal qRT-PCR

Total bacterial genomic DNA (gDNA) was isolated from mouse cecal content using the Quick-DNA Fecal/Microbe MiniPrep Kit (Zymo Research, Irvine, CA, USA) according to manufacturer’s instructions. Isolated gDNA was quantified using Nano Drop ND1000 Spectrophotometer. Real time PCR amplification was conducted with the QuantStudio 5 analyzer using 5 ng gDNA per reaction, PowerUp SYBR Green Master Mix (Applied Biosystems, Waltham, MA) and 0.5 µM primers; sequences are listed in Table 1. Relative abundance of target bacteria to the total bacteria was determined using the Comparative threshold (CT) method. Data depicts fold changes relative to saline treated mice (controls).

### 2.5. Tissue qRT-PCR

Total RNA from the proximal colon and ileum was extracted using RNeasy Plus Universal Mini Kit (Qiagen, Hilden, Germany) according to manufacturer’s instructions. Isolated RNA was quantified using Nano Drop ND1000 Spectrophotometer. A total of 2 ug of the total RNA was reverse transcribed using SuperScript IV VILO (Invitrogen, Waltham, MA, USA). Real time PCR amplification was conducted with a QuantStudio 5 analyzer using PowerUp SYBR Green Master Mix (Applied Biosystems, Waltham, MA, USA) and 1 µM primers, sequences listed in Table 2. Relative expression of target genes to the housekeeping gene glyceraldehyde 3-phosphate (GAPDH) was determined using the Comparative threshold (CT) method. Data depicts fold changes relative to saline treated mice (controls).

### 2.6. Enzyme-Linked Immunoassay (ELISA)

Plasma and cecal IgA levels were measured by ELISA. Cecal content was weighed, suspended at a 50 mg feces/mL concentration in sterile PBS, and vortexed at 800 rpm overnight at 4 °C. The fecal suspension was then centrifuged at 14,800 rpm for 15 min at 4 °C and the supernatant was collected. The cecal supernatant was diluted from 1:1–1:10 as needed, mouse plasma was diluted 1:1000, and the IgA levels were measured using the Mouse IgA Uncoated ELISA kit (Invitrogen, Waltham, MA, USA) according to manufacturer’s instructions. Mouse serum was collected and used to measure Total Iron Binding Capacities (TIBC) level using the Total Iron-Binding Capacity (TIBC) Assay Kit (BIOVision, Milpitas, CA, USA) according to manufacturer’s instructions.

### 2.7. Immunohistochemistry (IHC)

Immunohistochemistry was performed as previously described [18]. Briefly, paraffin embedded tissue sections were de-paraffinized using SafeClear, 100% ethanol, 95% ethanol, and 70% ethanol, followed by Tris-EDTA antigen retrieval. Slides were then washed three times with phosphate buffered saline with 0.1% triton (PBS-T) for 10 min, and blocked with 10% donkey serum in PBS-T for one hour at room temperature. Sections were then incubated with rabbit anti-CD3 overnight at 4 °C. The next day slides were washed three times with PBS-T, and incubated in the dark for 1 h at room temperature with the secondary antibody, anti-rabbit Alexa 568, as well as Anti-Dapi for nuclear staining. All antibodies were diluted in blocking solution. Slides were again washed three times with PBS-T and mounted with Fluoromount-G™ (Electron Microscopy Sciences, Hartfield, PA, USA). Images were acquired using a Keyence BZ-X810 Fluorescent Microscope and Keyence BZ-X800 Viewer software. All parameters for image acquisition were kept constant and three images of each section in similar locations and were captured with 20 × magnification. Images were semi-quantified using Image Pro+ 7.0 (Media Cybernetics Bethesda, MD, USA). For each image, a color file was created for each specific hue of interest with the same intensity and saturation. Object size was manually selected and object count was collected. In a similar manner, the sum of the entire area of the tissue was collected. Both object count and area sum were then exported to Excel for analysis.

### 2.8. Statistics

All statistical analysis were performed using GraphPad Prism^®^ 9 software (San Diego, CA, USA). All data represents the mean ± the standard error from the mean (SEM). Significance between normally distributed groups was calculated using a two tailed *t*-test and variances between multiple groups was determined by one way ANOVA with a Tukey post hoc test. The threshold for statistical significance was set at *p* ≤ 0.05.

## 3. Results

### 3.1. Bodyweight and Intestinal Assessment

**Figure 1:**
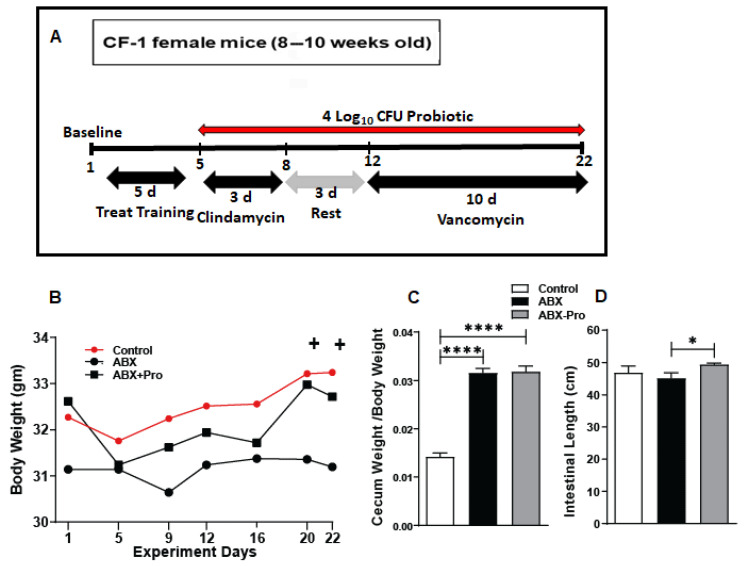
Mouse model and its tolerance. (**A**) Mice were treated with the probiotic blend starting on day five until study end. To induce gut dysbiosis, starting on day five animals were treated with clindamycin for 3 days; control mice received saline. Beginning on day 12, mice were treated with either vancomycin or saline for 10 days, after which they were euthanized on day 22 and intestine was dissected. (**B**) Mice were weighed at specific time points and compared. (**C**) Cecum was weighed and compared to mouse body weight. (**D**) Intestinal length from proximal duodenum to rectum was measured and compared. Values represent mean ± standard error of the mean. + *p* = 0.06, * *p* < 0.05, **** *p* < 0.0001.

CF-1 mice were treated as depicted in Figure 1A and their bodyweight was monitored at distinct time points throughout the trial. At baseline animals in each treatment group had similar body weights. Throughout the study, control and antibiotic + probiotic (ABX-PRO)-treated animals steadily gained weight, however the weight of the antibiotic + saline (ABX)-treated animals remained stable at baseline levels (Figure 1B). Altered gut microbial abundance causes increased cecum weight [19]. Thus, as a surrogate marker in alterations in antibiotic-induced bacterial abundance, we measured cecum-to-body weight. As expected, we saw a significant increase in cecum-to-body weight in all mice treated with antibiotics (Figure 1C). Additionally, mouse intestinal length was longer in the ABX-PRO group compared to the ABX group (*p* = 0.037) (Figure 1D) suggesting that probiotics protected against the blunting of intestinal absorptive capacity induced by antibiotics.

### 3.2. Gut Microbiota Analysis

**Figure 2:**
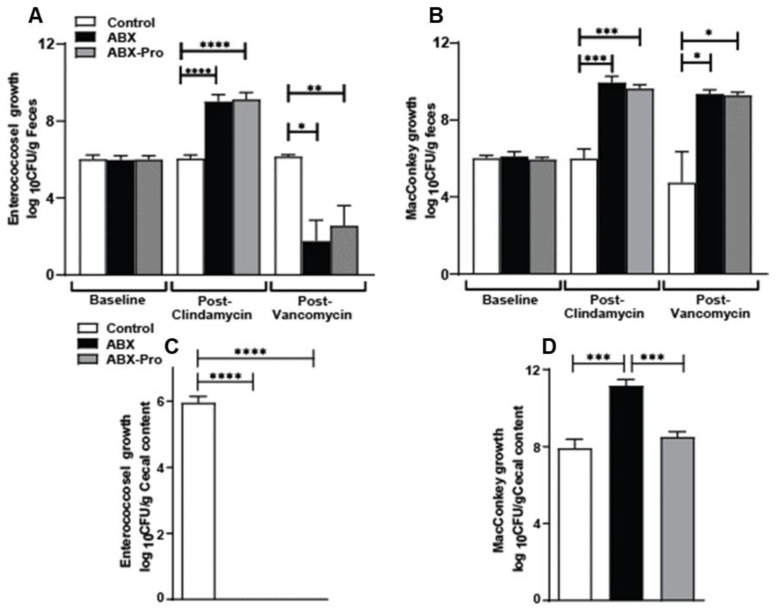
Bacterial Culture: Mice were treated as outlined in Figure 1. At specific time points fecal pellets were collected, and at euthanasia cecum was dissected and both specimens underwent selective agar plating for: (**A**) Enterococcoci in fecal pellets; (**B**) aerobic and facultative anaerobic gram-negative rods bacteria in fecal pellets. (**C**) Enterococcoci in cecal content; and (**D**) aerobic and facultative anaerobic gram-negative rods in cecal content. Values represent the mean Log_10_ Colony Forming Units (CFU)/gram ± standard error of the mean. * *p* < 0.05, ** *p* < 0.01, *** *p* < 0.001, **** *p* < 0.0001.

At distinct time points throughout the trial fecal pellets were collected and underwent selective bacterial agar plating to detect changes in growth of aerobic, facultative anaerobic gram-negative rods and enterococcoci. Following 3 days treatment with clindamycin, both antibiotic treated groups showed an expansion in enterococcoci (*p* = 0.0001) and gram-negative rods (*p* = 0.0001) (Figure 2A,B). Vancomycin treatment for 10-days depleted enterococci in both ABX (*p* = 0.009) and ABX-PRO (*p* = 0.019) groups (Figure 2A); and as expected, gram-negative bacteria were unaffected by vancomycin treatment (Figure 2B). Following euthanasia, the cecum was immediately dissected and its contents were plated on selective agar. Supporting the fecal pellet data, no enterococci growth was detected in either antibiotic-treated groups (Figure 2C). However, ABX mice had significant overgrowth gram-negative rods compared to the control (*p* = 0.0005) and ABX-PRO mice (*p* = 0.0002) (Figure 2D).

**Figure 3:**
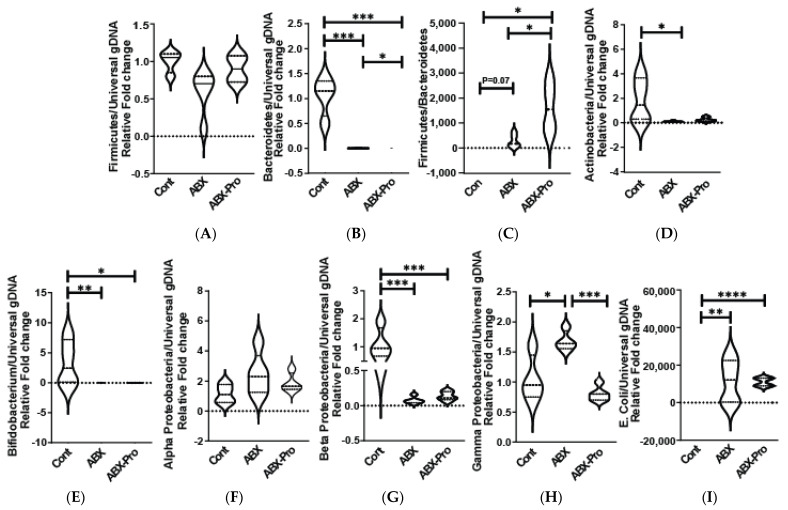
Cecal Microbiome: Mice were treated as described in Figure 1. At euthanasia, cecum was dissected and contents were collected, genomic DNA (gDNA) was isolated, and qRT-PCR was utilized to detect the relative abundance of (**A**) Firmicutes and (**B**) Bacteroidetes. (**C**) The ratio of the relative abundance of Firmicutes to Bacteroidetes was calculated. Further qRT-PCR was preformed to determine the relative abundance of (**D**) Actinobacteria, (**E**) Bifidobacterium, (**F**) Alphaproteobacteria (**G**) Betaproteobacteria, (**H**) Gammaproteobacteria, and (**I**) *E. coli*. Values represent mean ± standard error of the mean. * *p* < 0.05, ** *p* < 0.01, *** *p* < 0.001, **** *p* < 0.0001.

To further assess intraluminal microbial changes observed in cecal plating, qRT-PCR was performed on extracted cecal bacterial genomic DNA (gDNA). The relative abundances of the phyla Firmicutes, Bacteroidetes, and Actinobacteria were measured. The ABX group trended towards a lower relative abundance of Firmicutes compared to control and ABX-PRO-treated animals (Figure 3A). Bacteroidetes were significantly decreased in both antibiotic treated groups relative to controls (*p* = 0.0001) (Figure 3B), but ABX mice had a significantly higher relative abundance of Bacteroidetes compared to ABX-PRO treated mice (*p* = 0.040) (Figure 3B). There was a significant increase in the Firmicutes to Bacteroidetes ratio of the relative fold change in the ABX-PRO mice compared to the control (*p* = 0.018) and ABX groups (*p* = 0.023) (Figure 3C). Actinobacteria decreased significantly in the ABX but not in the ABX-PRO group (Figure 3D). Bifidobacterium, a gut commensal genus of Actinobacteria, was measured to investigate the variation found in abundance of Actinobacteria in the ABX group. Both groups of antibiotic-treated mice had a significant loss in Bifidobacterium compared to controls. (Figure 3E). Proteobacteria is a phylum containing many opportunistic bacteria. In order to elucidate the antibiotic-induced gram-negative bacteria overgrowth, changes in three classes of Proteobacteria were assessed. While no significant changes occurred between treatment groups for alphaproteobacteria (Figure 3F), betaproteobacteria abundance was decreased in both antibiotic treatment groups compared to controls (Figure 3G); and there was a significantly higher abundance of Gammaproteobacteria in ABX mice compared to control and ABX-PRO-treated mice (Figure 3H). However, *Escherichia coli* (*E. coli*), a species of Gammaproteobacteria, was significantly elevated in both antibiotic treatment groups compared to controls (Figure 3I).

### 3.3. Gut Microbe and Host Immune Responses

**Figure 4:**
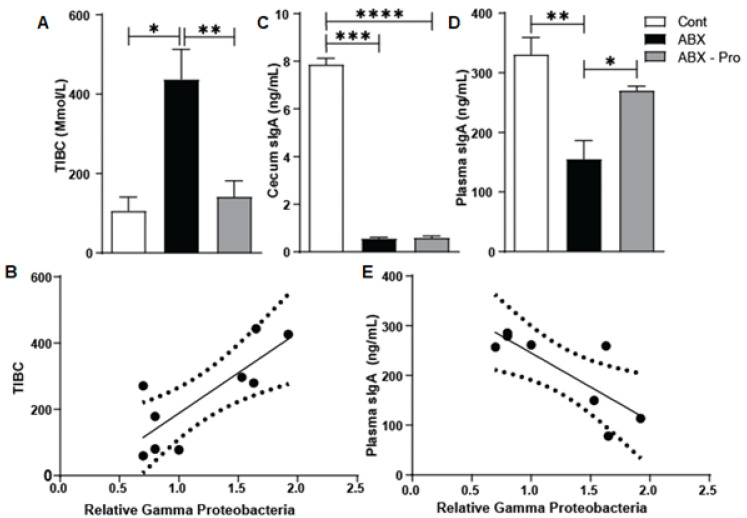
Effects of Gammaproteobacteria Overgrowth: Animals were treated as described in Figure 1. At euthanasia the cecum was dissected, and blood was collected and serum and plasma were isolated. (**A**) TIBC was measured in mouse serum by ELISA. (**B**) The correlation between relative Gammaproteobacteria abundance in mouse cecum to the TIBC was calculated. R^2^ = 0.65, *p* = 0.008. The sIgA in mouse (**C**) cecal content and (**D**) mouse plasma was measured by ELISA. (**E**) The correlation between relative Gammaproteobacteria abundance in mouse cecum to sIgA in mouse plasma was calculated. R^2^ = 0.625, *p* = 0.02. Values represent mean ± standard error of the mean. * *p* < 0.05, ** *p* < 0.01, *** *p* < 0.001, **** *p* < 0.0001.

As a means to enhance growth and survivability, pathogens attempt to acquire iron from the host. Previously we found that the probiotic blend exhibited nutritional immunity properties in mice exposed to *C. difficile* [15]. To determine if the probiotic blend had an effect on nutritional immunity, we measured the total iron binding capacity (TIBC) in the serum collected at euthanasia. The ABX group had elevated TIBC (*p* = 0.02) but this was mitigated in the ABX-PRO-treated mice. (*p* = 0.001) (Figure 4A). To establish a potential cause for the elevation of TIBC in antibiotic-treated animals the relative growth abundance of gammaproteobacteria in cecum contents to serum TIBC levels was correlated. This identified a positive correlation between TIBC and the relative abundance of gammaproteobacteria (*p* = 0.002) (Figure 4B).

Immunoglobulin IgA is an antibody induced by antigens, both food and microbial; and secretory IgA (sIgA) can protect against toxins and infections [20]. To determine if antibiotic exposure with or without the probiotic blend altered host immune responses, unbound sIgA levels were assessed in the cecal contents (Figure 4C) and plasma (Figure 4D). In the cecum all antibiotic-treated mice had significantly lower unbound sIgA levels compared to controls. However, ABX mice also had significantly lower plasma sIgA levels compared to control and ABX-PRO treated mice. Additionally, an inverse correlation was found between the relative abundance of plasma sIgA and cecal gammaproteobacteria (*p* = 0.019) (Figure 4E).

### 3.4. Vitamin A Metabolism

Animals are not able to synthesize vitamin A de novo, making it an essential dietary nutrient. The species *Bacillus indicus HU36™* produces the carotenoids lycopene, astaxanthin, lutein, and β-carotene, a precursor to retinoic acid [16]. In the intestine vitamin A and its carotenoid derivatives are metabolized to form transcriptionally active *all-trans* retinoic acid (*at*RA) or to form retinyl esters that are stored in the liver. The tissue concentration of *at*RA is determined by strict temporal and spatial regulation of genes involved with its metabolism. *All-trans* retinoic acid assists the host with intestinal immune responses. To evaluate the effect of antibiotics with or without the probiotic blend on the host’s ability to produce *at*RA, the mRNA expression of a panel of enzymes responsible for vitamin A metabolism and storage were assessed in the ileum and proximal colon.

**Figure 5:**
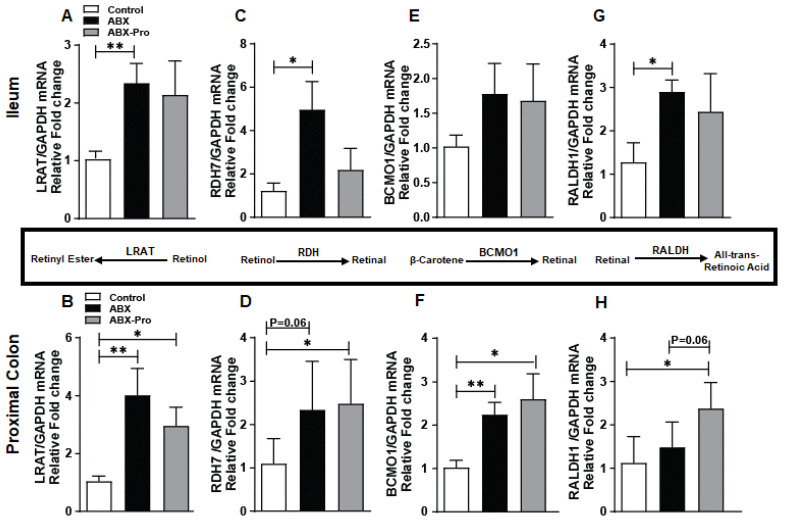
Vitamin A Metabolism. Mice were treated as described in Figure 1. At euthanasia, ileum and proximal colon were dissected and relative mRNA expression of (**A**,**B**) LRAT, (**C**,**D**) RDH7, (**E**,**F**) BCMO1, and (**G**,**H**) RALDH1 was detected by qRT-PCR. Values represent mean fold change from control mice ± standard error of the mean. * *p* < 0.05, ** *p* < 0.01.

Retinyl esters, the most abundant form of vitamin A in the body, are synthesized by the enzyme Lecithin:retinol acetyltransferase (LRAT) which causes transesterification of retinol absorbed from diet or produced from the reduction of retinal [21]. In the ileum LRAT mRNA expression was increased in the ABX group (*p* = 0.005) compared to controls (Figure 5A). Comparatively, in the proximal colon LRAT mRNA expression was increased in the ABX (*p* = 0.006) and ABX-PRO treated mice (*p* = 0.18) relative to controls (Figure 5B).

The enzyme retinol dehydrogenase 7 (RDH7) converts retinol to retinal. In the ileum, RDH7 mRNA expression was elevated in the ABX group compared to controls (*p* = 0.021) and ABX-PRO mice (Figure 5C). However, in the proximal colon both ABX (*p* = 0.034) and ABX-PRO mice (*p* = 0.064) had increased RDH7 mRNA expression compared to controls (Figure 5D).

βeta-Carotene 15, 15′monooxegnase 1 (BCMO1) cleaves diet acquired β-Carotene into two molecules of retinal [22]. While an upward trend of BCMO1 mRNA expression was found in the ileum for both antibiotic-treated groups (Figure 5E), in the proximal colon this induction was significant in both groups of antibiotic-treated mice compared to controls (Figure 5F). Retinaldehyde dehydrogenases 1 (RALDH1) converts retinal to *at*RA. In the ileum RALDH1 mRNA expression was increased in antibiotic only-treated mice compared to controls (*p* = 0.032) (Figure 5G). However, in the proximal colon, ABX-PRO mice had higher mRNA expression compared to controls (*p* = 0.038) and mice treated with antibiotics only (*p* = 0.064) (Figure 5H).

**Figure 6:**
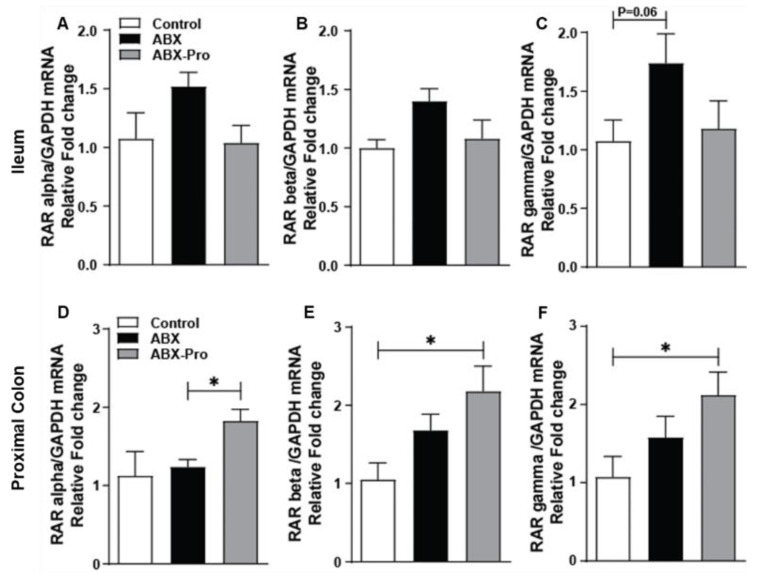
Retinoic Acid Receptors. Mice were treated as described in Figure 1. At euthanasia, ileum and proximal colon were dissected and relative mRNA expression of RAR-alpha, RAR-beta and RAR-gamma in the (**A**–**C**) Ileum and the (**D**–**F**) Proximal Colon was detected using qRT-PCR. Values represent mean ± standard error of the mean. * *p* < 0.05.

Expression of retinoic acid receptors (RAR)-alpha, -beta, and -gamma were measured in order to infer relative levels of *at*RA. In the ileum the mRNA expression of RAR-alpha, -beta, and -gamma trended higher in the ABX group compared to control and ABX-PRO groups (Figure 6A–C). In the proximal colon the ABX-PRO mice had elevated mRNA expression of all three RARs (Figure 6D–F). Specifically, ABX-PRO treated mice had increased expression of RAR alpha compared to the ABX group (*p* = 0.015) (Figure 6D), and increased mRNA expression compared to control mice for RAR-beta (*p* = 0.031) (Figure 6E) and RAR-gamma (*p* = 0.038) (Figure 6F).

### 3.5. Serum Amyloid

**Figure 7:**
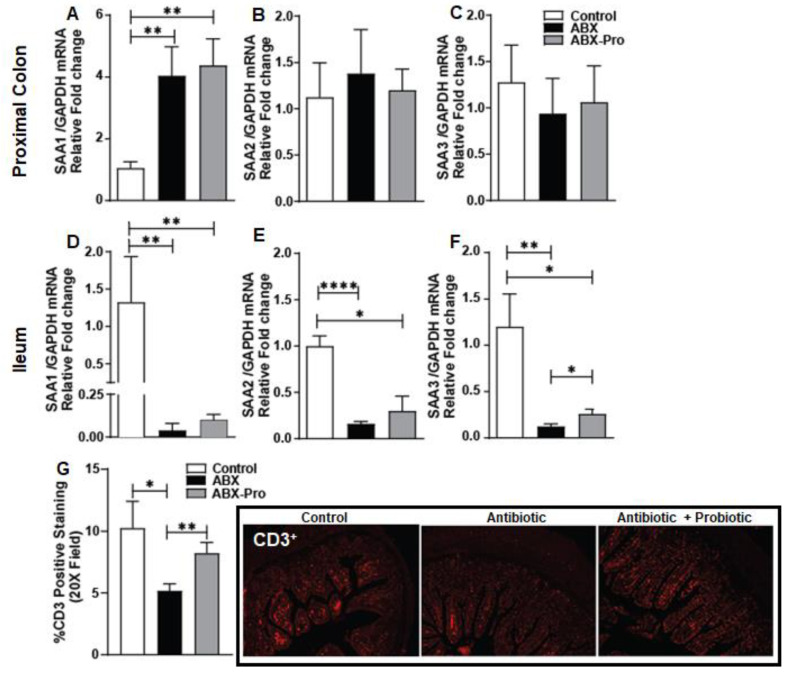
Serum Amyloid A and Immune Function. Mice were treated as described in Figure 1. At euthanasia, proximal colon and ileum were dissected and used to prepare RNA or fixed in formalin and embedded in paraffin. Relative mRNA expression of SAA-1, SAA-2, and SAA-3 in the (**A**–**C**) Proximal Colon and ileum (**D**–**F**) was detected by qRT-PCR. (**G**) CD3 was visualized by immunohistochemistry in sections of ileum fixed in formalin. Total object count was divided by total staining area to determine the percent CD3+ cells for each treatment group. All images were acquired using a 20× objective. Images are representative of at least replicate images captured per mouse in four mice per treatment group. Values represent mean ± standard error of the mean. * *p* < 0.05, ** *p* < 0.01, **** *p* < 0.0001.

Serum amyloid A (SAA) is a family of immunoregulatory retinol binding proteins. These proteins bind retinol from the intestinal epithelium and the liver and circulate it during bacterial infection [8]. To further investigate the effects of the probiotic blend on intestinal immunity the mRNA expression of SAA-1, -2, and -3 was measured. In the proximal colon, both antibiotic groups showed increased mRNA expression of SAA-1 compared to controls (Figure 7A); however, there were no differences in the expression of SAA-2 and SAA-3 between treatment groups (Figure 7B,C). In the ileum, while both antibiotic treatment groups had significant losses in mRNA expression of SAA-1, SAA-2, and SAA-3 compared to control animals (Figure 7D–F), SAA-3 mRNA expression was greater in ABX-PRO mice compared to ABX mice (*p* = 0.03). (Figure 7F).

Serum amyloid A assists in the upregulation of T cells which modulate antimicrobial defenses and subsequent gut microbial composition [23]. Because changes in SAA in the proximal colon and ileum were found, the abundance of CD3^+^ T in these intestinal regions was assessed by immunohistochemistry (Figure 7). In the ileum of the ABX group, positive staining for CD3^+^ T cell was decreased to nearly half the abundance of the control and ABX-PRO groups (Figure 7G), suggesting a loss of total T cells present in the small intestine with antibiotic treatment. The same analysis was completed in the proximal colon but no difference was noted in the CD3^+^ T cells between groups (data not shown).

### 3.6. Inflammatory Markers

**Figure 8:**
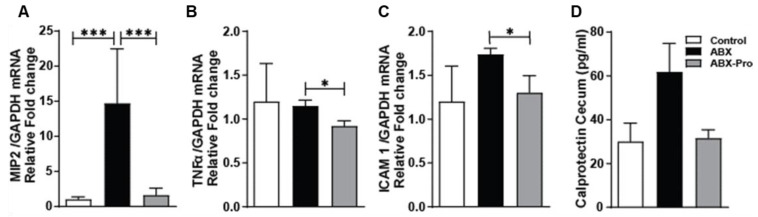
Intestinal Inflammation. Mice were treated as described in Fig1. At euthanasia, proximal colon was dissected and relative expression of (**A**) MIP2, (**B**) TNFα, and (**C**) ICAM1 mRNA was detected by qRT-PCR. Cecum was dissected and (**D**) cecal content was used to measure calprotectin by ELISA. Values represent mean ± standard error of the mean. * *p* < 0.05, *** *p* < 0.001.

To determine if changes in gut microbial composition and immune responses could affect intestinal inflammation, a panel of inflammatory markers was tested. In the proximal colon the mRNA expression of macrophage inflammatory protein 2 (MIP2) was significantly elevated in the ABX group compared to the control (*p* < 0.0001) and ABX-PRO groups (*p* = 0.0007) (Figure 8A), and the mRNA expression of tumor necrosis factor alpha (TNFα) (Figure 8B) and Intercellular Adhesion Molecule 1 (ICAM 1) (Figure 8C) was significantly lower in the ABX-PRO compared to the ABX group. Fecal calprotectin is used as a biomarker for intestinal inflammation [24]. The ABX group had elevated protein levels of calprotectin in their cecal content compared to both control and ABX-PRO groups (Figure 8D). These data suggest the probiotic blend protected against antibiotic-induced intestinal inflammation.

## 4. Discussion

Short-term antibiotic treatment causes the gut microbiota to shift to a long-term perturbed state, one which alters the functionality of the gut microbiota including pathogen resistance [6]. Here, we find that in addition to disrupting gut microbial abundance, several host-derived immune mechanisms are also impacted by antibiotic exposure. Importantly, the data presented reveal prophylactic supplementation with a spore-forming, non-pathogenic *Bacillus* species-containing probiotic blend mitigates certain aspects of intestinal-derived immune responses altered by antibiotic exposure.

Vancomycin is a gram-positive targeting antibiotic that only affects gut microbes when taken orally. Clindamycin has broad anaerobic bacteria coverage, but does not have activity against aerobic gram-negative bacteria or enterococci [25]. Our data support these antibiotic activities in mice, and while we saw no evidence of the probiotic blend protecting against losses in gram-positive commensals, co-supplementation with the probiotic prevented antibiotic-induced overgrowth of Gammaproteobacteria, a predominantly pathogenic class of gram-negative bacteria. This coincided with preservation of circulating sIgA levels, mitigation of SAA mRNA expression and maintenance of T cell abundance in the ileum, and reduction in markers of inflammation in the proximal colon. Thus, these data suggest the probiotic blend supports host immune responses that are altered with antibiotic therapy.

Comparison of the Firmicute to Bacteroidetes ratio is often used to characterize gut microbiota and host health [26]. Studies suggest that in addition to a skewed ratio being a marker of gut dysbiosis, an increased ratio correlates with obesity and a decreased ratio correlates with inflammatory conditions such as Crohn’s Disease and colitis [26]. In this study we found the ABX-PRO group had an increased Firmicute to Bacteroidetes ratio. While antibiotics have been suggested to contribute to the development of obesity, this has mainly been attributed to antibiotics provided early in life and repeated exposure for ≥3 courses of antibiotics [27]. However, the prior reported factors do not apply to our model, the weight gain in the ABX-PRO group was similar to control mice, and ABX-PRO mice were protected against other factors that are associated with obesity such as low-grade intestinal inflammation. Thus, the increased Firmicute:Bacteroidetes ratio may be a result of the mice receiving the Bacillus species-containing probiotic which falls within the Firmicutes phylum.

In addition to mechanisms supported by the gut microbiota, the host has several defenses to protect itself against pathogens. As a first line of intestinal defense, sIgA directly binds to pathogenic bacteria and prevents its translocation through the epithelium. A study in neonatal mice found sIgA deficiency correlated with an overabundance of Gammaproteobacteria, leaving the mice more susceptible to intestinal damage [28,29]. Our data corroborate these findings where we found a negative correlation with plasma sIgA and cecal Gammaproteobacteria relative abundance.

The secretion of IgA is regulated by *at*RA, which induces B cell-IgA secretion and class switching [29]. Here we found mice treated with the probiotic blend had increased colonic mRNA expression of a panel of enzymes that metabolize vitamin A to *at*RA and *at*RA receptors. As *a*tRA is typically thought to be metabolized in the small intestine, we speculate the spectrum of carotenoids produced by *B. indicus* HU36™, a *Bacillus* species included in the probiotic blend, may have contributed to this induction in the proximal colon. As a result, there would be increased T and B cells homing and IgA production. To support this notion, we found mice treated with the probiotic blend had rescued SAA mRNA expression in the ileum compared to mice treated with only antibiotics. SAA is an acute phase inflammatory protein that transports retinol to myeloid cells. In response to retinol binding to LDL receptor-related protein 1 (LRP1) the myeloid cells metabolize the retinol into *at*RA [30]. The atRA then promotes B and T cell homing resulting in increased production of IgA and increased protection from enteric infection [30]. Previous studies have shown animals lacking LRP1 are unable to develop adaptive immune responses following pathogenic infection [30]. This suggests mice treated with only antibiotics would have compromised adaptive immune responses due to losses in SAA mRNA expression in the ileum. This was confirmed with our finding that mice treated only with antibiotics had lower abundance of CD3^+^ T cells in the ileum, and these responses coincided with increased markers of intestinal inflammation.

## 5. Conclusions

In conclusion, supplementation with a spore-forming *Bacillus*-species probiotic blend protected against antibiotic-induced gut dysbiosis and preserved key immune response properties employed by the host to counteract overgrowth of opportunistic microbes and intestinal inflammation. As antibiotic therapy is necessary for bacterial infections, it is important to find ways for the host to protect itself against its negative effects. Our study has limitations; in particular, we did not perform whole metagenomic shotgun sequencing of fecal and cecal samples. Further studies incorporating technologies such as metagenomic shotgun sequencing that are able to take a deeper dive into microbial strain diversity as well as genetic content and function are warranted to delineate key mechanisms of each *Bacillus*-species in the probiotic blend, whether they work in concert or individually, as well as optimizing treatment doses and timing of delivery with antibiotic therapy [31]. Additionally, incorporation of transcriptomics as with RNA-Seq analysis of tissue samples could assist with determining causal effects of microbial changes on host responses [32].

## Figures and Tables

**Table 1:** Bacterial primer sequences.

Target	Type	Sequence
Universal	Total	ACTCCTACGGGAGGCAGCAG
		ATTACCGCGGCTGCTGG
Actinobacteria	Phyla	CGCGGCCTATCAGCTTGTTG
		ATTACCGCGGCTGCTGG
Bacteroidetes	Phyla	GGCGACCGGCGCACGGG
		GRCCTTCCTCTCAGAACCC
Firmicutes	Phyla	GGAGYATGTGGTTTAATTCGAAGCA
		AGCTGACGACAACCATGCAC
Alphaproteobacteria	Class	ACTCCTACGGGAGGCAGCAG
		TCTACGRATTTCACCYCTAC
Betaproteobacteria	Class	CCGCACAGTTGGCGAGATGA
		CGACAGTTATGACGCCCTCC
Gammaproteobacteria	Class	GAGTTTGATCATGGCTCA
		GTATTACCGCGGCTGCTG
Bifidobacterium	Genus	GGGTGGTAATGCCGGATG
		TAAGCCATGGACTTTCACACC
*Escherichia coli*	Species	GTGTGATATCTACCCGCTTCGC
		AGAACGCTTTGTGGTTAATCAGGA

**Table 2:** Gene Primer Sequences.

Target	Primer	Sequence
Β-Carotene 15, 15′ Monooxygenase 1	BCMO1 F	GAGCAAGTACAACCATTGGT
	BCMO1 R	AACTCAGACACCACGATTC
Glyceraldehyde-3-Phosphate Dehydrogenase	GAPDH F	AGGTCGGTGTGAACGGATTTG
	GAPDH R	TGTAGACCATGTAGTTGAGGTCA
Intercellular Adhesion Molecule 1	ICAM1 F	GTGATGCTCAGGTATCCATCCA
	ICAM1 R	CACAGTTCTCAAAGCACAGCG
Macrophage Inflammatory Protein	MIP2 F	GCGCCCAGACAGAAGTCATAG
	MIP2 R	AGCCTTGCCTTTGTTCAGTAT C
Lecithin Retinol Acyltransferase	LRAT F	GCAGTTGGGACTGACTCCAT
	LRAT R	GCAGTTGGGACTGACTCCAT
Retinoic Acid Receptor-alpha	RARα F	AGAGCAGCAGTTCCGAAGAG
	RARα R	AGTGGTAGCCGGATGATTTG
Retinoic Acid Receptor-beta	RARβ F	AATGCCACCTCTCATTCAGG
	RARβ R	GTCTGCAACAGCTGGAAATG
Retinoic Acid Receptor-gamma	RARƴ F	CCACCAAATGCATCATCAAG
	RARƴ R	ATCCGCAGCATTAGGATGTC
Retinoic Aldehyde Dehydrogenase 1	RALDH1 F	ATACTTGTCGGATTTAGGAGGCT
	RALDH1 R	GGGCCTATCTTCCAAATGAACA
Retinol Dehydrogenase 7	RDH7 F	CCTGGCGGGTTCAGGACTA
	RDH7 R	CGAGGATGTCTGGTCCCAC
Serum Amyloid A 1	SAA1 F	ATCACCAGATCTGCCCAGGA
	SAA1 R	CCTTGGAAAGCCTCGTGAAC
Serum Amyloid A 2	SAA2 F	ACCAGATCTGCCCAGGAGAC
	SAA2 R	GCATGGAAGTATTTGTCTCCATCT
Serum Amyloid A 3	SAA3 F	GACATGTGGCGAGCCTACTC
	SAA3 R	TTGGCAAACTGGTCAGCTCT

F: forward; R: reverse.

## Data Availability

Data supporting reported results can be requested in writing to the corresponding author.
